# Community-level variability in Bronx COVID-19 hospitalizations associated with differing population immunity during the second year of the pandemic

**DOI:** 10.1093/ve/veae090

**Published:** 2024-11-01

**Authors:** Ryan Forster, Anthony Griffen, Johanna P Daily, Libusha Kelly

**Affiliations:** Department of Systems and Computational Biology, Albert Einstein College of Medicine, Bronx, NY 10461, United States; Department of Cell Biology, Albert Einstein College of Medicine, Bronx, NY 10461, United States; Department of Microbiology & Immunology, Albert Einstein College of Medicine, Bronx, NY 10461, United States; Department of Medicine (Infectious Diseases), Albert Einstein College of Medicine, Bronx, NY 10461, United States; Department of Systems and Computational Biology, Albert Einstein College of Medicine, Bronx, NY 10461, United States; Department of Microbiology & Immunology, Albert Einstein College of Medicine, Bronx, NY 10461, United States

**Keywords:** genomics, epidemiology, modeling, transmission, immunity and local

## Abstract

The Bronx, New York, exhibited unique peaks in the number of coronavirus disease 2019 (COVID-19) cases and hospitalizations compared to national trends. To determine which features of the severe acute respiratory syndrome coronavirus 2 (SARS-CoV-2) virus might underpin this local disease epidemiology, we conducted a comprehensive analysis of the genomic epidemiology of the four dominant strains of SARS-CoV-2 (Alpha, Iota, Delta, and Omicron) responsible for COVID-19 cases in the Bronx between March 2020 and January 2023. Genomic analysis revealed similar viral fitness for Alpha and Iota variants in the Bronx despite nationwide data showing higher cases of Alpha. However, Delta and Omicron variants had increased fitness within the borough. While the transmission dynamics of most variants in the Bronx corresponded with mutational fitness-based predictions of transmissibility, the Delta variant presented as an exception. Epidemiological modeling confirms Delta’s advantages of higher transmissibility in Manhattan and Queens, but not the Bronx; wastewater analysis suggests underdetection of cases in the Bronx. The Alpha variant had slightly faster growth but a lower carrying capacity compared to Iota and Delta in all four boroughs, suggesting stronger limitations on Alpha’s growth in New York City (NYC). The founder effect of Iota varied between higher vaccinated and lower vaccinated boroughs with longer delay, shorter duration, and lower fitness of the Alpha variant in lower vaccinated boroughs. Amino acid changes in T-cell and antibody epitopes revealed Delta and Iota having larger antigenic variability and antigenic profiles distant from local previously circulating lineages compared to Alpha. In concert with transmission modeling, our data suggest that the limited spread of Alpha may be due to a lack of adaptation to immunity in NYC. Overall, our study demonstrates that localized analyses and integration of orthogonal community-level datasets can provide key insights into the mechanisms of transmission and immunity patterns associated with regional COVID-19 incidence and disease severity that may be missed when analyzing broader datasets.

## Introduction

The Bronx, New York, a borough of New York City (NYC), was an early epicenter of SARS-CoV-2 transmission during the early period of the pandemic (CDC 2022). The cumulative rate of confirmed cases of COVID-19 in the Bronx as of 11 September 2022 was 11 495 cases/100 000 individuals. The Bronx has 1 472 654 residents, it is a densely populated region, with approximately 34 920 people/sq. mile, and has an ethnically and racially diverse population. It is the third highest county by population density and the 13th highest in overall population within the U.S. Of the NYC boroughs, the Bronx had the highest hospitalization and death rate due to COVID-19: 1752 hospitalizations/100 000 individuals and 470 deaths/100 000 individuals over the 3 years of the pandemic. Several factors likely contributed to these high rates. Residents of the Bronx experience high rates of asthma and other chronic respiratory ailments, which are known risk factors for severe COVID-19 (CDC 2020, United States Census Bureau 2021). Furthermore, certain demographic groups are severely impacted by COVID-19, including Black/African American, Native American, Asian, or Hispanic/Latino individuals, as well as those of low socioeconomic status. More than 50% of the Bronx population are Black/African American or Hispanic/Latino descent (CDC 2020) and 24% of residents are below the poverty line. Taken together, the Bronx is home to a uniquely vulnerable population to SARS-CoV-2 infection and severe COVID-19.

To understand the local determinants of SARS-CoV-2 infection, we previously investigated the viral diversity of SARS-CoV-2 in the Bronx during the first year of the pandemic and found that SARS-CoV-2 viral diversity in the Bronx matched global trends, except for the emergence of a Bronx-originating lineage (Fels et al. 2022). Chronic infection of immunocompromised individuals by SARS-CoV-2 has been observed in the Bronx previously (Fels et al. 2022), has been linked to an accelerated mutation rate, and is proposed to be a mechanism responsible for the emergence of variants of concern (VOCs; Kemp 2021, Chaguza 2023), making local surveillance efforts in vulnerable patient populations such as the Bronx critical. Genomic surveillance during the second year of the pandemic conducted in New York State (NYS) identified a unique viral lineage: B.1.526, the New York–originating Iota variant of interest (VOI). Iota was the dominating lineage in NYS in contrast to the lineage dominating the Western world at the time: B.1.1.7, the Alpha VOC. The lineages B.1.1.7 and B.1.526 were not preferentially represented in vaccine breakthrough patients, compared to unvaccinated patients infected during the same sampling period, implying that the SARS-Cov-2 vaccine-induced immunity against each lineage was similar (Duerr et al. 2021). Despite this observation, estimates of transmissibility suggested that Alpha was more transmissible than Iota, and these studies were either limited to the genomes selected or investigated over larger geographic regions (Annavajhala et al. 2021, Petrone Me et al. 2022, Dellicour et al. 2023). As the pandemic has progressed, new VOCs have emerged, and a greater understanding of how immune escape mutations and single nucleotide positions facilitate transmission has developed (Wright et al. 2022, Obermeyer et al. 2022).

We observed that the Bronx had unique peaks and trends in COVID-19 cases, hospitalizations, and dynamics of VOI and VOC compared to national data and local adjacent communities. To determine which features of the SARS-CoV-2 virus and population health data might explain these differences, we characterized the longitudinal genomic epidemiology of SARS-CoV-2 in the Bronx from March 2020 until January 2023. We quantified viral dynamics, mutational fitness, antibody escape, T-cell epitope changes, and transmissibility and analyzed how these are associated with large-scale population health data. Our studies provide a comprehensive assessment of how different viral variants interacted with the Bronx population over time. This multifaceted approach reveals intricate interactions between competing SARS-CoV-2 variants and the immune landscape in the Bronx, highlighting the critical role of local surveillance for tailoring regional public health strategies and interventions against constantly evolving pandemic dynamics.

## Results

### The Bronx deviates from US aggregate data and other boroughs during the second year of the SARS-CoV-2 pandemic in COVID-19 hospitalization rates

**Figure 1.**
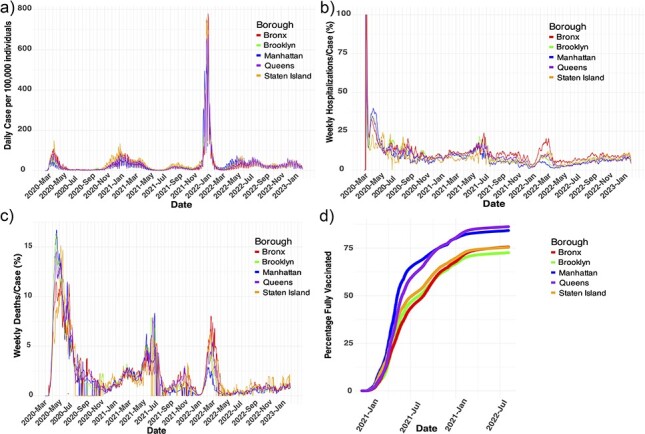
Bronx COVID-19 hospitalizations are the highest relative to other NYC boroughs during the second year of the pandemic, with the lowest vaccination rate. (a) A plot of SARS-CoV-2 PCR-diagnosed cases per 100 000 individuals in the Bronx compared with the other NYC boroughs from March 2020 to February 2023. (b) Percent hospitalizations and (c) deaths per case in the five boroughs were plotted weekly over the same timeline to account for lag in reporting. (d) Percentage of individuals in the five boroughs who received a complete series of vaccination over time.

Trends in case rates and hospitalizations resulting from SARS-CoV-2 infection significantly differed in the Bronx when compared with US aggregate data (Supplementary Fig. S1a and b). Bronx case rates, measured per 100 000 individuals defined by positive SARS-CoV-2 Polymerase Chain Reaction (PCR) tests, show five peaks over the 3 years of the pandemic. Wastewater PCR testing for the SARS-CoV-2 *N* gene complements patient COVID-19 rates and reveals a community viral load mirroring major trends in case rates. Four out of five peaks in the Bronx’s COVID-19 cases surpass the Bronx’s average case rate for the entire pandemic, except for the summer of 2021, where the peak falls below the 99% confidence interval. However, the community viral load measured via wastewater remains above the 99% confidence interval for the same time period (Supplementary Fig. S1a). When comparing the Bronx to the other four NYC boroughs, it strongly matches its neighboring communities (mean pairwise Pearson correlation = 0.9692), with Staten Island having the highest case rate over the course of the pandemic (Fig. 1a).

Notably, Bronx hospitalizations are below average for the summer of 2021, in contrast to the US aggregate, which remains above average during the same period (Supplementary Fig. S1b). While hospitalization counts provide a clear picture of case trajectories, the autocorrelation function adds value by quantifying the regularity and periodicity of these hospitalization patterns over time. This allows for a statistical comparison of wave dynamics, revealing how closely subsequent peaks, such as Omicron, mirror the initial outbreak in terms of timing and scale. The Bronx peaks in winter cases had statistically significant autocorrelation in hospitalizations and cases, with the strongest correlation between the original introduction of SARS-CoV-2 and the winter of 2021 (Supplementary Fig. S1c). To understand variability nationwide during the 3 years of the pandemic, we further analyzed state-level data across the USA splitting by state and standardizing hospitalizations, variations above and below the state averages are most evident during the summer of 2021, with consistent trends observed during the winter peaks and inconsistency during the spring and summer months (Supplementary Fig. 1d). The spring of 2021 is associated with dominance of Iota and the summer of 2021 is associated with the arrival, then dominance of the Delta VOC, which is considered a more virulent strain (Carabelli et al. 2023, Dellicour et al. 2023), yet there were fewer cases and hospitalizations relative to previous peaks within the Bronx (Supplementary Fig. 1a and b). When we employed a robust linear model, incorporating cases, hospitalizations, and an interaction term for the dominating VOC, the analysis revealed no statistical difference in the slope of the regressions between the periods dominated by the Delta and Iota variants during the second year (Supplementary Fig. S1e). Despite the equivalent number of hospitalizations between the Delta and Iota peaks, the Bronx had the greatest number of hospitalizations per case compared to other boroughs during the second year and least correlated trends (Fig. 1b, pairwise average Pearson correlation = 0.7649). Mortality per case was similar between boroughs (pairwise average Pearson correlation = 0.8917) and overall matched trends in hospitalizations per case (Fig. 1c).

### Genomic analysis demonstrates that Bronx Alpha and Iota VOC had equivalent fitness and Delta had increased mutational fitness

**Figure 2.**
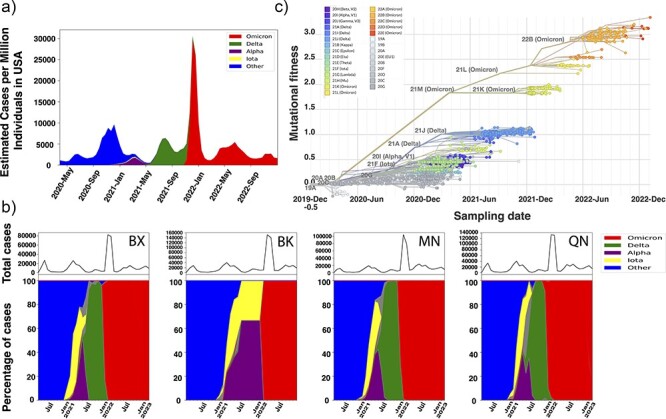
Alpha and Iota are quickly replaced with Delta clades during the second year of the pandemic due to a higher globally defined relative fitness. (a) Estimated proportion of cases by major VOCs/VOIs in the USA colored by VOC/VOI. (b) A regression plot representative of 2791 Bronx (BX) sequences as points connected by phylogeny, mutational fitness is plotted on *y*-axis against sampling time on the *x*-axis. Mutational fitness derived from a globally trained hierarchical Bayesian multinomial logistic regression model using PyRo (Obermeyer et al. 2022). It quantifies the relative transmission fitness of sequences based on their combinations of mutations associated with transmission advantage relative to the Wuhan reference sequence. (c) The relative proportion of Omicron, Delta, Alpha, Iota, and non-World Health Organization (WHO) variant lineages over time in the BX (*n* = 2791), Brooklyn (BK; *n* =1038), Manhattan (MN; *n* =41 710), and Queens (QN; *n* = 2151).

While case rates were correlated during the pandemic, the Bronx diverged from the national average and other boroughs in variant dynamics. The USA had a relatively large estimated caseload of Delta compared to Alpha and a relatively smaller Iota outbreak during the second year (Fig. 2a). The Bronx experienced a different trend, where the Iota variant took up a larger proportion of cases than Alpha or Delta (Supplementary Fig. S2a). Overall 12 VOCs were identified in the Bronx; only four are well represented in the sampling process. These are, in chronological order of appearance in the Bronx, Iota, Alpha, Delta, and Omicron, each with a higher number of mutations compared to the preceding VOC (Supplementary Fig. 2b). We find a similar trend in NYC’s other boroughs, even when increasing the sampling depth from 2791 samples from the Bronx to 41 710 sequences from Manhattan. Only four of the five boroughs had sufficient sampling, and therefore Staten Island was excluded from genomic analyses. The relative turnover of variants reveals similar patterns, cocirculating Iota and Alpha are replaced with Delta, and Omicron then overshadows Delta. The only exception is Brooklyn where there was very little sampling of Delta (Fig. 2b). Given the relatively higher caseload of Iota compared to Delta, we initially assessed transmission fitness of sampled viral strains through the Nextstrain pipeline’s pretrained Bayesian multinomial logistic regression model. This machine-learning model quantifies relative fitness to the Wuhan strain by quantifying the number of mutations the model has learned to associate with continued transmission at a global scale (Obermeyer et al. 2022). Despite the higher mutation count in Bronx Alpha sequences compared to Iota (Supplementary Fig. S2b), the mutational fitness of Alpha and Iota was found to be identical. Conversely, Delta and Omicron exhibited an increase in fitness. The preceding first-year lineages had the lowest mutational fitness and the highest number of pangolin lineages (Fig. 2c, Supplementary Fig. S3). However, the arrival of Iota in the Bronx was associated with the highest pairwise nucleotide differences, which ultimately dropped along with the arrival of Delta (Supplementary Fig. S4).

### Estimation of transmission parameters of Iota, Alpha, and Delta in the Bronx, Brooklyn, Manhattan, and Queens

**Figure 3.**
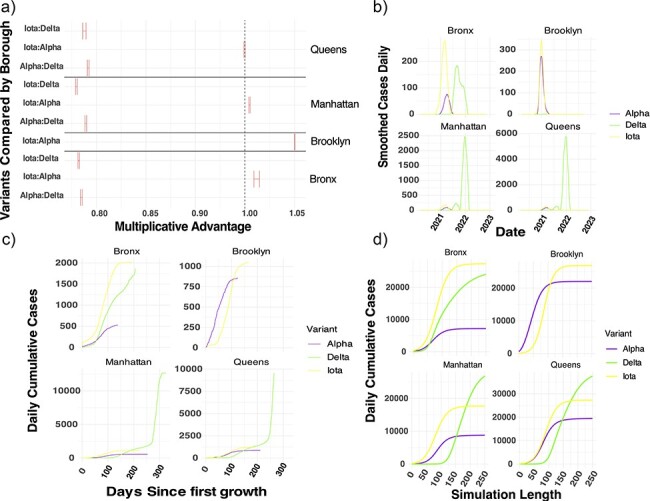
Fitting the turnover and estimated case data of Alpha, Iota, and Delta during the second year to find relative fitness and absolute fitness advantages between variants. (a) A forest plot comparing the relative fitness of variants by borough, the estimates of MA come from training a local PyRo logistic regression model on the local variant proportions over time from Fig. 2. (b) Variant proportion data were mapped onto case data, smoothed and daily smoothed cases were used to visualize more closely the variant dynamics during the second year in each borough. A logistic model was fitted onto (c) plotted cumulative cases during the second year aligned on the initial detection for close visual inspection and comparison of dynamics. (d) The fitted Bayesian model is plotted above as well for each borough-variant comparison.

**Figure 4.**
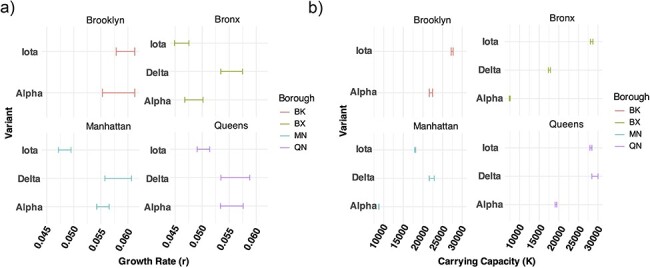
Parameter values of (a) growth rate and (b) carrying capacity for logistic growth dynamics by borough and variant. 95% confidence intervals are plotted to assess uncertainty in parameter estimates. BX, Bronx; BK, Brooklyn; MN, Manhattan; QN, Queens.

To verify the results of the globally trained mutational fitness model, we constructed a Bayesian logistic regression model to estimate the rate of replacement between variants using the proportion of variants over time. Rates were compared between Alpha, Iota, and Delta by calculating multiplicative advantage (MA) for the Bronx, Brooklyn, Queens, and Manhattan independently. The MA between Iota and Alpha hovered around one, indicating that the rate of disappearance of Iota was roughly equal to the rate of growth of Alpha. The MA of either Iota or Alpha compared to Delta was below one highlighting an abrupt change in lineage turnover to Delta. The MA for variants varied between the boroughs. Iota and Alpha’s MA estimate increased by borough in the order of Queens, Manhattan, Bronx, and Brooklyn (Fig. 3a). The logistic regression of viral variant proportions does not explicitly model case dynamics and therefore does include epidemiological context. Therefore, we mapped the variant proportion data onto cases and found that Iota had higher daily new and cumulative infections than Alpha and Delta in the Bronx. In the highly vaccinated boroughs, Manhattan and Queens, Delta cases were delayed, but higher than Iota and Alpha (Fig. 3b and C). Cumulative cases resembled logistic growth curves for all variants in all boroughs, Delta appears to come in multiple waves reflecting either double logistic growth in the Bronx or exponential growth in the second waves of Delta in Manhattan and Queens (Fig. 3c). Given that the initial phases appear logistic, we fit a logistic growth equation to each variant’s cumulative growth curve to estimate absolute fitness of each variant in each borough. Overall, the fit models captured the dynamics well (pseudo-*R*^2^ = 0.99 for all models); however, the parameter estimates are wider than the model using variant proportions only. In Brooklyn and the Bronx, Iota has statistically similar growth rate estimates. In the Bronx, Delta has statistically higher growth rate estimates. In Manhattan and Queens, both Alpha and Delta have statistically higher growth rate estimates (Fig. 4a). Despite differences in growth rates by borough, the carrying capacity of Iota was always higher than Alpha, and only in the Bronx was Iota’s carrying capacity significantly higher than Delta’s (Fig. 4b). Duration of the Iota wave was higher relative to the Alpha wave in the Bronx and Brooklyn, while reversed for Manhattan and Queens, which had overall longer duration when considering both variants. Iota’s arrival in all of the boroughs was consistently prior to Alpha’s, with first detection in the Bronx, Manhattan, Queens, and then Brooklyn (Supplementary Fig. S5a and b).

### WHO variant’s antigenic distances from preceding lineages differ by borough

**Figure 5.**
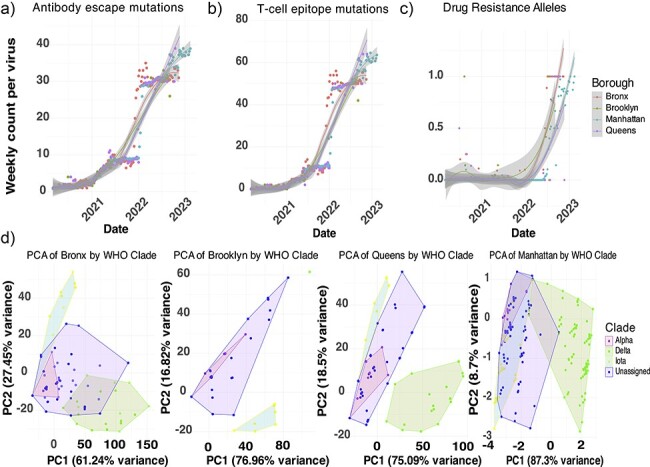
The number of immune epitopes per virus over time is consistent between boroughs, but variant epitope diversity varies respective to prior circulating lineages of SARS-CoV-2. (a) Antibody epitope, (b) T-cell epitope, and (c) drug-resistance alleles detected weekly per virus over the course of the pandemic across Bronx, Brooklyn, Manhattan, and Queens. Trends estimated using LOESS regression for nonlinear trends. (d) PCA dimensionality of pairwise hamming distances of antibody epitope mutation profiles of Alpha, Iota, Delta, and previously circulating lineages of SARS-CoV-2 for the Bronx (*n* = 2285 sequences), Brooklyn (*n* = 1001 sequences), Manhattan (*n* = 24 461 sequences), and Queens (*n* = 1910 sequences).

Given the split in vaccination coverage, severity, and variant dynamics during the second year between Bronx and Brooklyn versus Manhattan and Queens, we hypothesized that there were differing immune pressures among the boroughs shaping trends. To investigate putative evidence of differing immunity, population-level data on the number of mutations in immune epitopes, viral mutations associated with monoclonal antibody resistance, and antiviral treatments sourced from the COVID-19 Genomics United-Kingdom Consortium (COG-UK) database were quantified. Trends over time suggest similar dynamics in the weekly average sampling of antibody-impacting, T-cell-impacting, and drug-resistance alleles for the four boroughs despite vastly different sampling depths between Manhattan and other boroughs (Fig. 5a, b, and c). T-cell epitopes accumulated faster than antibody escape mutations, with most occurring with the arrival of Omicron (Fig. 5a and b). When examining antiviral resistance markers toward reduction of IC50 for Ensovibep (Angiotensin-converting enzyme-2-binding inhibitor), Nirmatrelvir (protease inhibitor), and Remdesivir (viral polymerase inhibitor) within the different VOC, we identified spurious occurrences of resistance mutations against Ensovibep within the spike gene and resistance mutations against Nirmatrelvir and Remdesivir within ORF1ab were identified in isolates of Iota, Delta, and Omicron, although not Alpha, most on average occurred later in the pandemic with Omicron (Fig. 5c). Epitope diversity differed within variants opposed to only between variants, Iota had a wider range of epitope changes per virus depending on the time it was sampled, with later sampled Iota strains having more. Conversely, Alpha had limited epitope diversity (Supplementary Figs S6 and S7). All VOC had statistically different numbers of antibody epitope changes per virus (*P* <3 × 10^−7^), with Alpha having the lowest number per virus (Supplementary Fig. S6). To quantify which epitopes were more similar to each other and how this differs between boroughs, we computed pairwise Hamming distances between sequences of Iota, Alpha, Delta, and previously circulating lineages as a measure of “antigenic distance.” Antigenic distances were visualized on a Principal Componet Analysis (PCA) for each borough with the greatest separation between all variants and Delta on the first principal axis (PC1) and Iota with preceding lineages on the second principal axis (PC2). A feature found in all boroughs is Alpha completely overlapping with preceding variants, while the explainable variance in PC2 decreases in Manhattan and Queens compared to Bronx and Brooklyn’s PC1 (Fig. 5d).

## Discussion

A major goal of SARS-CoV-2 surveillance is to uncover generalizable trends in the rates of COVID-19 cases and hospitalizations to inform public health strategies, yet it remains unclear what level of geographical granularity is most informative. Here, we show that in the Bronx, deviations from national trends were evident in the second year of the pandemic. These deviations reveal VOC interactions and patient outcomes that differ from those observed at larger geographic scales and highlight the need for local surveillance. We defined the transmission dynamics of Delta, Iota, and Alpha, which were the major sampled variants during the spring and summer of the second year of the pandemic. (1) We highlight three specific features of the SARS-CoV-2 pandemic in the second year that differentiate the Bronx and the other four boroughs from the USA: the dominance of Iota in place of the Alpha variant in early 2021, lower-than-average hospitalizations associated with the Delta variant in the same year, and higher-than-average hospitalizations associated with the introduction of the Omicron variant. (2) Features that differentiate the Bronx from the other boroughs were an increased number of hospitalizations per case, a lower vaccination rate, and differing dynamics of Iota and Delta variants’ relative carrying capacities.

One feature distinguishing COVID-19 VOC dynamics in NYC was the dominance of the VOC Iota at a time when Alpha was the dominant strain of the Western world at the time of cocirculation and was described to have superior binding efficiency to human Angiotensin-converting enzyme-2 (ACE-2) receptors (Carabelli et al. 2023). We explored whether Iota prevented the dominance of Alpha in the Bronx. We determined that Iota and Alpha had similar mutational fitness profiles for global spread as predicted by PyR_o_, and our Bayesian logistic regression model trained on local data suggests a similar trend in relative fitness locally. In terms of absolute fitness estimates, the logistic growth model of estimated case data highlights that across boroughs Alpha had the lowest carrying capacity compared to Iota and Delta. Alpha had a slightly higher growth rate, but with fewer cumulative cases. We hypothesized that Iota was less impacted by immunity and control measures across boroughs. Iota could have possessed a broader array of immune escape mechanisms due to strain variability, and others have noted greater resistance (higher IC50) to vaccinated sera than Alpha (Annavajhala et al. 2021, Choi et al. 2021). We found that boroughs with lower vaccination rates (Bronx and Brooklyn) had higher local relative fitness of Iota compared to Alpha when compared with boroughs with higher vaccination rates (Manhattan and Queens). This observation was backed by the relative duration of Iota and Alpha, where the Alpha wave lasted longer than the Iota wave in boroughs with higher vaccination rates and the opposite is true for boroughs with lower vaccination rates. The higher vaccinated boroughs had a longer duration of both variants overall (Supplementary Fig. S5). We might expect the opposite finding for a strain with more immune escape mutations, like Iota, to have greater fitness and duration in vaccinated communities, but higher heterogeneity between Iota variants and lower overlap of Iota variants with preceding lineages in less vaccine-immune populations could explain this observation (Fig. 5d). This suggests the combination of lineage diversity prior to Alpha and Iota, combined with the level of vaccination shaped borough-level competition between Iota and Alpha. Our findings also indicate that there is greater variability in T-cell epitopes in Iota Bronx strains than previously reported in Iota, which could be a result of sampling bias. However, temporal sampling of immune escape mutations suggests statistically similar trends in quantifying functionally relevant mutations across boroughs with vastly different sampling depths (Fig. 5a**–**c). Limited epidemiological studies suggest that these two VOCs had equivalent immune escape capacities with equal vaccine breakthrough infection rates (Duerr et al. 2021). Other studies have suggested that Alpha has had very few antigenic escape mechanisms that support our observations in the Bronx and the rest of NYC (García-Beltrán et al. 2021, Brown et al. 2021, Newman et al. 2022). Overall, the epitope observations in conjunction with logistic growth modeling (Fig. 2a) suggest that there was a trade-off between an increased carrying capacity in Iota and modest increases in the growth rate of Alpha infections resulting in an equivalent fitness as predicted by the PyR₀ mutation fitness model and the local logistic regression model (Figs 2c and 3a).

Our local study encompasses all reported, high-quality, sequences from the Bronx, Brooklyn, Manhattan, and Queens providing a more comprehensive perspective on the dynamics of Alpha and Iota in this region. There was a clear bias in the sampling between boroughs, with Manhattan having 10 times the sampling depth. Brooklyn had a lack of Delta sampled in Year 2 of the pandemic; however, the representation of Alpha and Iota was sufficient to analyze trends of these two variants in Year 2 (Fig. 2b). Our observations support a roughly equal overall fitness between Alpha and Iota within NYC and variations between boroughs. We suggest that the pre-existing establishment of Iota impacted the spread of Alpha in all boroughs, but pre-existing immunity determined by both vaccination and the lineage diversity present prior to both Iota and Alpha impacted their relative fitness and duration between boroughs. The temporal emergence patterns between boroughs suggest that the observed Iota founder effect was stronger in the unvaccinated boroughs compared to vaccinated boroughs, where there was a longer delay between the arrivals of Iota and Alpha (Supplementary Fig. S5a). The aggregated data from the USA suggest, on average, that the dynamics have played out differently elsewhere because of Alpha’s known mechanisms of increased transmission speed via more efficient ACE-2 binding (Carabelli et al. 2023).

We next turned to the distinctive pattern in NYC, the variation of the Delta peak across boroughs and over time. In the Bronx, the Delta peak in the summer of 2021 fell below the 99% confidence interval for mean Bronx cases despite a higher-than-average community viral load, as revealed by wastewater PCR testing for the SARS-CoV-2 N gene (Supplementary Fig. S1a). Given that there was little variant subdiversity at this point and continued detection of the virus, hospitalizations in the Bronx are below average during the summer of 2021, diverging sharply from the national aggregate, which remains above average during the same period (Fig. 1b). At this same time, deaths per case were low across boroughs. Together, these observations support a hypothesis, suggesting that fewer people were symptomatic or seeking treatment because their cases were less severe, resulting in lower-than-average COVID-19 cases and hospitalizations despite above-average SARS-CoV-2 wastewater detection. The observation that Delta does not have higher hospitalizations per case goes against the conventional argument that Delta was a more virulent VOC. Hospitalization and case data instead suggest that the community-level impact of hospitalization per case of Delta was equivalent to the preceding peak of mainly Iota and Alpha cases.

In contrast with Alpha and Iota, Delta was infecting hosts with greater immunity against SARS-CoV-2, as the population became more vaccinated or developed natural immunity from prior infections. Immunity in the hosts should reduce severity and possibly reduce its spread among the Bronx patient population. Given that the Bronx had the highest severity and the lowest vaccination rate, we would expect Delta’s growth to be more unchecked; however, we observed the opposite effect, where the Bronx’s Delta variant had the lowest carrying capacity compared to other boroughs during the initial growth phase. We should consider that Delta had undetected spread through the Bronx, which could explain why its carrying capacity was lower. Delta had advantages over other VOCs such as an increased mutational fitness, a greater number of immune escape mutations, and the largest antigenic distance from preceding lineages suggesting a greater ability to spread among immune populations. Essentially, the equal community-level virulence of Delta relative to previous variants could be linked to growing immunity within the Bronx, while the increased severity compared to other boroughs arises from Delta’s increased transmissibility and undetected spread. To be certain, better population immunity measures, such as seropositivity, are needed to verify this.

Finally, we focused on differences in hospitalizations in the Bronx during Omicron’s prevalence, which were higher than its time-series average (Supplementary Fig. S1b). The differences between the Bronx and other boroughs were greatest during the arrival of Omicron, which is likely due to similar phenomena described for Delta (Fig. 1b & Supplementary Fig. S1e). State-wide hospitalizations due to COVID-19 peaks were normalized and overlapped in December 2021 and were above each time-series average too, suggesting a period of uniformity in Omicron trends nationwide (Supplementary Fig. S1d). Within the Bronx, there was a significant correlation between Bronx Omicron cases and hospitalizations associated with the first introduction of SARS-CoV-2, suggesting similarities to the naive introduction observed at the start of the pandemic and could represent waning immunity to Omicron lineages (Stein et al. 2023). Evidence from Hong Kong suggests that Omicron sublineage BA.2 displays a hospitalization frequency similar to that of first-wave variants (Mefsin et al. 2022), which aligns with our autocorrelation analysis based on the Bronx population (Supplementary Fig. S1c).

In conclusion, our integrated analysis of SARS-CoV-2 and COVID-19 cases and hospitalization trends in the Bronx over the second year of the pandemic underscores the critical importance of localized studies in understanding the intricacies of viral dynamics. The deviations observed in the Bronx from national trends and local trends, such as the dominance of the Iota variant over Alpha in early 2021 and both the reduction of hospitalizations associated with the Delta variant and varying carrying capacity by borough, reveal the need for a more granular examination of the pandemic’s impact in local areas. The distinctive pattern in case rates, the discrepancy between diagnosed cases and community viral load, and the complex interplay between hospitalization trends and viral variants add layers of complexity to the local dynamics. The observed dynamics between Alpha and Iota in the Bronx may not universally apply beyond the local region, emphasizing the importance of localized studies in unraveling the complexities of the pandemic. Overall, our analysis highlights localized factors influencing viral dynamics that may be missed in larger, countrywide, or global studies, providing a foundation for tailored local public health interventions.

## Materials and methods

### Public health data

All NYC COVID-19 epidemiological data, which includes diagnosed cases, hospitalizations, deaths, and testing rates, come from the NYC Department of Health and Mental Hygiene (https://github.com/nychealth). All community health measures for COVID-19 are defined by the most up-to-date definitions by the Centers for Disease Control and Prevention (CDC)’s Council of State and Territorial Epidemiologists (Coronavirus Disease 2019 (COVID-19) 2021 Case Definition|CDC 2021). Wastewater community SARS-CoV-2 viral loads are based on quantitative PCR copies of the viral nucleocapsid gene per liter of water sampled from two locations in the Bronx: Hunts Point and Ward’s Island (https://data.cityofnewyork.us/Health/). Data for trends in the estimated VOC cases in the USA were curated from the Global Initiative on Sharing All Influenza Data (GISAID): https://gisaid.org, John Hopkins COVID-19 Data Repository: https://github.com/CSSEGISandData/COVID-19, and Covariants https://covariants.org/cases, while the US aggregate hospitalizations and state hospitalizations were obtained from the CDC’s COVID-NET database: https://www.cdc.gov/coronavirus/2019-ncov/covid-data/covid-net/purpose-methods.html.

### Time-series statistics

All scripts were custom and written in Python. Stationarity, a behavior describing the independence of the variance and mean from time, was assessed to measure a constant baseline. The augmented Dicker–Fulley test checked for stationary behavior and normality assumption was assessed with the Shapiro–Wilk test (*P* > .05). All time series were considered stationary, meaning they have a constant mean and variance over time, and normalized time-series means were used to compare the relative magnitude of cases or hospitalizations.

The autocorrelation analysis was conducted using the pandas autocorrelation_plot function in Python. This function generates a plot of the autocorrelation coefficients of the time series, which can be used to identify any patterns or trends in the data over time. The autocorrelation coefficient is a measure of the correlation between a variable and its lagged values.

The autocorrelation coefficient of lag *k* can be calculated using the following formula:


$${\rho _k} = {{{{\mathop \sum }}_{t = k + 1}^n\left( {{y_t} - \bar y{\mathrm{}}} \right)\left( {{y_{t - k}} - {\mathrm{}}\bar y} \right)} \over {{{\mathop \sum }}_{t = 1}^n{{({y_t} - \bar y{\mathrm{}})}^2}}}\\[5pt]$$

where *y* is the time-series variable, *n* is the sample size, and *k* is the lag. To calculate the confidence interval, the function uses the following formula:


$$confidence{\ }interval = \pm {\ }\frac{{z - \alpha }}{2}*standard\_error$$

where $\frac{{z - \alpha }}{2}$ is the critical value for the normal distribution at the${\ }\frac{\alpha }{2}$ significance level (where alpha is the significance level), and standard_error is the standard error of the autocorrelation estimate. The standard error is estimated as follows:


$$standard\_error = \sqrt {\frac{{\left( {1 + n\_lags} \right)}}{{n\_obs}}} $$

where *n*_lags is the number of lags being plotted and *n*_obs is the total number of observations in the time series.

To measure the correlation between daily hospitalizations and daily cases, values ranging from January 1, 2021 to October 31, 2021 were selected for the periods where Iota dominated and the peak in cases where only Delta was detected (no Omicron detected). The MASS package was used to create a robust linear model with an interaction term for the dominating VOC and the cases and the *F*-test was used to test the significance of that term’s influence on the daily hospitalizations: $H\sim{\beta _0} + {\mathrm{ }}\beta C + \beta C*V{\mathrm{ }}$, where $H$ = hospitalizations, $C$ = cases, and $V$ = dominating VOC.

Finally, linear correlations between boroughs were assessed using Pearson correlations via the R stat package.

### Genomic data

The GISAID was used to collect 2792 Bronx (10.55876/gis8.240811wq), 1001 Brooklyn (10.55876/gis8.240811zu), 41 710 Manhattan (10.55876/gis8.240811xa), and 2151 Queens (10.55876/gis8.240811vt) SARS-CoV-2 sequences and associated metadata. The GISAID database (Shu and McCauley 2017) is an open-access platform that facilitates the sharing of viral sequences and associated metadata for research and public health purposes. The sequences were filtered for high-quality sequences based on GISAID’s quality control criteria. WHO variant designation and lineages was assigned via the Nextclade commandline tool (Aksamentov et al. 2021, Rambaut et al. 2020).

### Nextstrain and PyRo analysis

The default Nextstrain pipeline (Hadfield et al. 2018) was used for molecular epidemiological analysis of the 2792 Bronx sequences. The pipeline involved several steps, including alignment, phylogenetic inference, and visualization. The fasta sequences were aligned using Multiple Alignment using Fast Fourier Transform (MAFFT) (Katoh 2002), maximum likelihood phylogenetic inference was then performed using IQ-TREE (Nguyen et al. 2014), and the resulting tree was visualized using the auspice web application (Hadfield et al. 2018). The pipeline was run using the Snakemake workflow management system, which is facilitated for reproducibility and scalability. Mutational fitness was estimated using PyRo, a hierarchical Bayesian multinomial logistic regression model that was pretrained on 6.4 million SARS-CoV-2 sequences. This model, integrated into the Nextstrain pipeline, quantifies the relative fitness of each viral sequence based on the combination of mutations it carries. The fitness estimation takes into account the global distribution of these mutations and their association with continued viral transmission. The hierarchical nature of the PyRo model allows for the inclusion of both lineage-specific and global effects, providing a robust estimate of how certain mutations contribute to the relative fitness of the virus in the context of its evolutionary history, and we ended up using this model to assess relative fitness of local strains in the context of global transmission (Obermeyer et al. 2022).

### Epidemiological modeling

We performed a Bayesian analysis using logistic growth models to evaluate the relative fitness of SARS-CoV-2 variants (Iota, Alpha, and Delta) across the Bronx, Brooklyn, Queens, and Manhattan. The model assumed that variants grew linearly in logit space, with growth rates and initial proportions assigned Normal(0, 1) priors. A 5.5-day generation time was applied. Stochastic variational inference was utilized, employing a mean-field AutoNormal guide and a ClippedAdam optimizer, with evidence lower bound (ELBO) loss minimized over 1501 iterations (Supplementary Fig. S8). The MA, representing relative fitness, was calculated by exponentiating the difference in relative rates between pairs of variants.

The analysis relied on several key assumptions: logistic growth in logit space, linear time dependence of variant growth, independence among variant lineages, and a multinomial likelihood for observed counts. Convergence was monitored through the ELBO loss curve, ensuring the robustness of the final model.

Alpha, Iota, and Delta proportions were multiplied by cases per day, and then kernel density was smoothed to get estimated cases contributed by each variant. From these data, we did two analyses: (I) we plotted the chronology and duration (Supplementary Figs S5) and (ii) fit a logistic growth model to each variant incorporating case data. We utilized a logistic growth model to examine cumulative COVID-19 cases for the SARS-CoV-2 variants Iota, Alpha, and Delta across the Bronx, Brooklyn, Queens, and Manhattan. The model, fitted using the nlsLM function in R, relied on initial parameter estimates derived from estimated case data for each borough-variant combination, with bounds to ensure realistic fits. The model assumed that cumulative cases follow a logistic growth pattern, with independent growth for each variant in each borough. Additionally, we assumed consistency in growth dynamics across boroughs, though with distinct parameters for each. Model performance was evaluated using pseudo-*R*^2^ values, and confidence intervals were calculated to ensure reliability. The model’s results were then used to simulate and visualize expected cumulative cases, highlighting the differences in growth dynamics across regions.

### Quantifying epitope mutations and drug resistance mutations

We utilized the alignments generated by the Nextstrain pipeline to annotate single amino acid changes relative to the Wuhan reference. To further investigate the potential implications of these amino acid changes, we utilized the COG-UK Mutation Explorer: https://sars2.cvr.gla.ac.uk/cog-uk/, a comprehensive SARS-CoV-2 database that focuses on observed amino acid replacements with antigenic roles in the human immune response. This database contains >2 million genome sequences specifically for UK SARS-CoV-2 data, which is curated and analyzed to track mutations of immunological importance that are accumulating in current VOCS and VOIs. These mutations have the potential to alter the neutralizing activity of monoclonal antibodies, convalescent sera, and vaccines, as well as changes in epitopes recognized by T-cells, including those with reduced T-cell binding. The database also includes mutations that have been shown to confer SARS-CoV-2 resistance to antiviral drugs (Wright et al. 2022). All analyses were made using custom Python scripts to count antibody resistance mutations, the antibodies impacted by them, the number of mutations in T-cell epitopes, and drug-resistance-associated mutations. Single amino acid polymorphisms (SAAPs) in the local Bronx sequences were called using the Nextclade pipeline and cross-referenced with the SAAPs in the COG-UK Mutation Explorer database to create a set of immune epitope changes for each sampled Bronx virus. A PCA was used to cluster antigenic relationships between individual viruses based on the immune epitope changes they share quantified in the form of hamming distances. To test for quantitative differences in epitopes, an ordinarily least squares model was used comparing each virus’s contribution to the variation in epitope mutations ($E$) over time. The following formula describes the model: $E\sim{\beta _0} + {\beta _{iota}}{V_{iota}} + {\beta _{alpha}}{\ }{V_{alpha}} + {\ }{\beta _{delta}}{V_{delta}} + {\beta _{omi}}{\ }{V_{omi}} + {\beta _{iota:time}}{V_{iota:time}}\ldots. + {\beta _{omi:time}}{V_{omi:time}}{\ }$

The $\beta $ values for each virus were compared pairwise via an *F*-test to obtain statistical significance.

## Supplementary Material

veae090_Supp

## Data Availability

The source code used for analysis and figure generation is hosted on GitHub at https://github.com/kellylab/SARS-CoV-2-surveillence.git.
