# Supplementary material for: Community-level variability in Bronx COVID-19 hospitalizations associated with differing population immunity during the second year of the pandemic
Source: Virus Evol. 2024 Nov 1;10(1):veae090. doi: 10.1093/ve/veae090 (PMC11604118; doi:10.1093/ve/veae090)
Supplement: veae090_Supp [file veae090_supp.zip › suppl_data/Supplemental_content_COVID_3yrs_Bronx-v4.docx]

**Supplementary materials:**


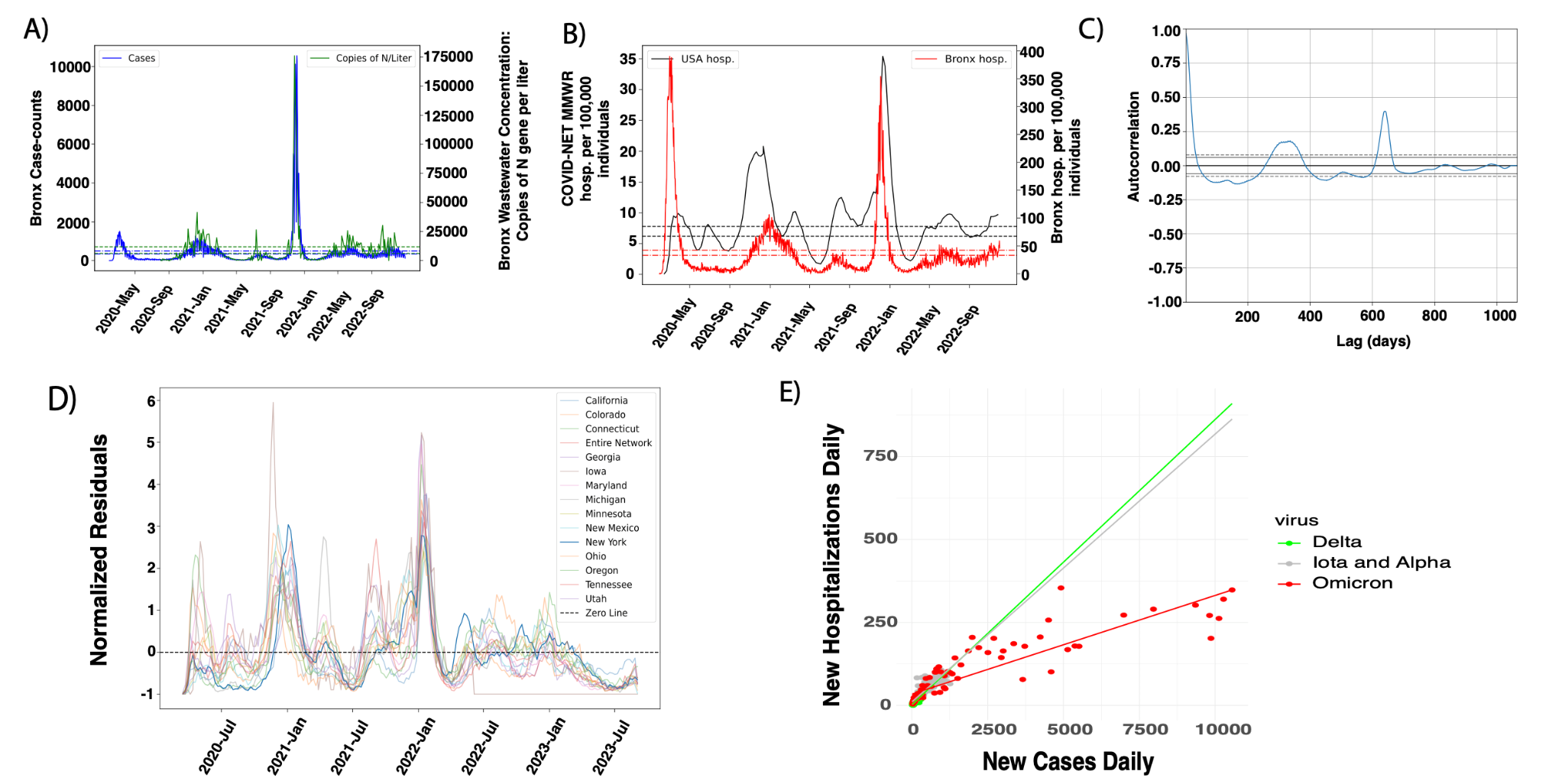


**Supplemental Figure 1: Bronx COVID-19 hospitalizations on average are below the U.S.A aggregate during year two of the pandemic, and the hospitalizations per case during the period of Delta VOC dominance are equivalent to the hospitalizations per case before it arrived in the Bronx. A)** A plot of SARS-CoV-2 PCR diagnosed cases per 100,000 individuals in the Bronx compared with wastewater quantification of SARS-CoV-2 community load via qPCR of the nucleocapsid protein. **B)** A plot of Bronx and U.S.A hospitalizations due to

COVID-19 per 100,000 individuals over time. The dotted lines indicate the 99% confidence interval values for a time series average any part of the time series above it is higher than average. **C)** Normalized COVID-19 hospitalizations by state were plotted and directly compared, variability above and below the zero line indicates different behavior during a given period of time. **D)** Autocorrelation of hospitalizations due to COVID-19 indicate 3 statistically correlated peaks across different days (lag), as they are correlation values above the 99% confidence interval (dotted line). The First peak in winter 2020 is at the beginning, then winter 2021 and winter 2022 as the winter peaks continue they appear to increase in magnitude. Omicron was first introduced in 2021 lagging more than 600 days after the initial peak in hospitalizations. **E)** A robust linear model of the relationship between cases and hospitalizations during the second year and accounted for the statistical interaction between dominating VOC and cases with no significant difference in new hospitalizations per new case during Delta VOC dominance,

p-value = 0.65.


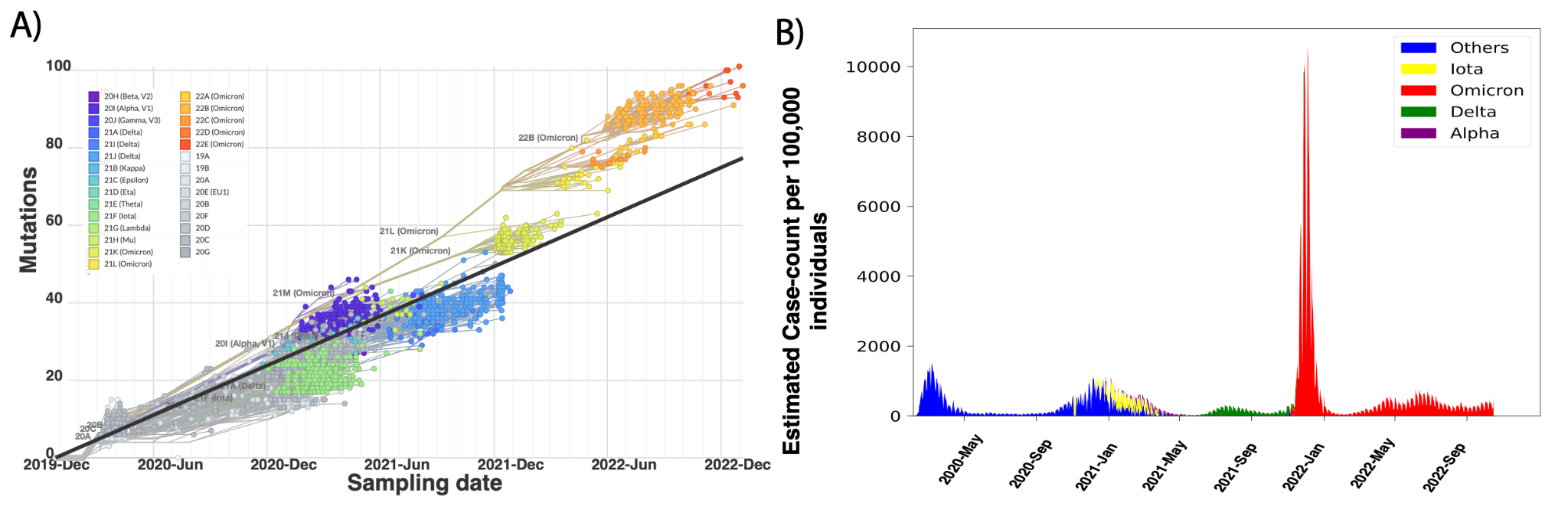


**Supplemental Figure 2: Mutation rate and variant proportions mapped onto case data from Bronx . A)** Mutation rate regression of 2,792 viruses sequenced in the Bronx, points are connected by phylogenetic order. On the x-axis is sampling date and on the y-axis is the number of nucleotide mutations. The mutation rate is 25 nucleotide substitutions a year relative to the Wuhan reference. **B)** Estimated proportion of cases by major VOC in the Bronx.


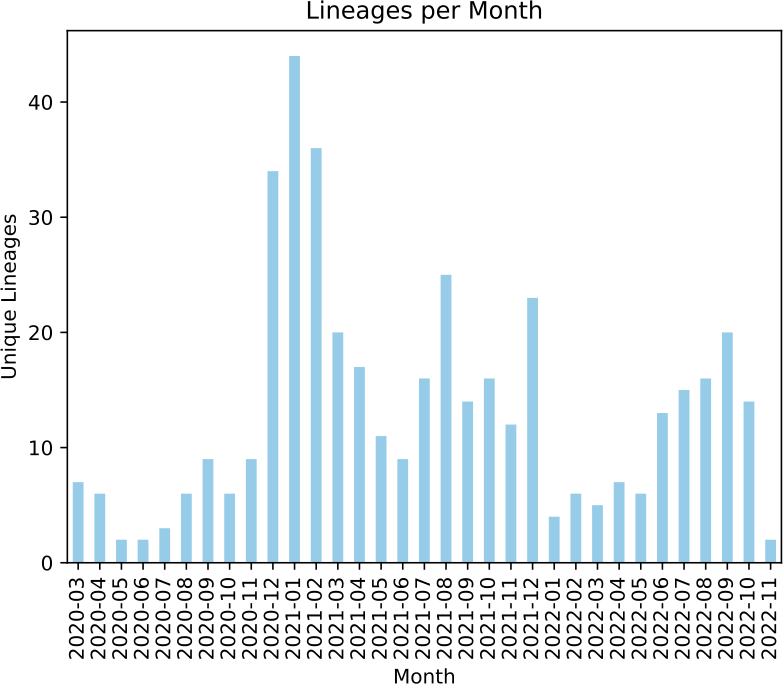


**Supplemental Figure 3:** Unique number of pangolin lineages per month. Pangolin lineages are geographic-dependent clustering of closely related viruses and represent a granular view of viral diversity. The peak in lineage diversity was observed in January 2021 during the initial introduction of vaccines in the Bronx.


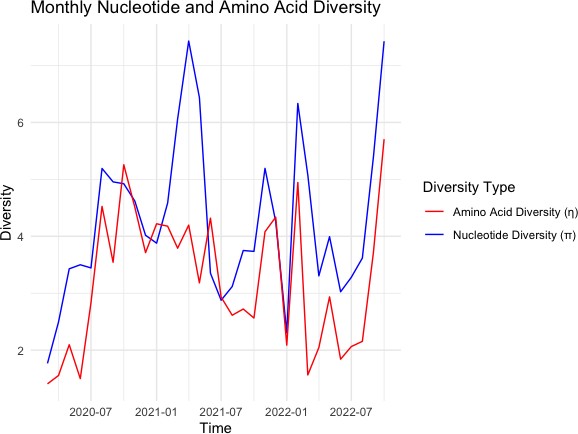


**Supplemental Figure 4:** Nucleotide diversity and amino acid diversity measured as the pairwise differences in residue changes between sequences sampled in the same month over the course of the pandemic.


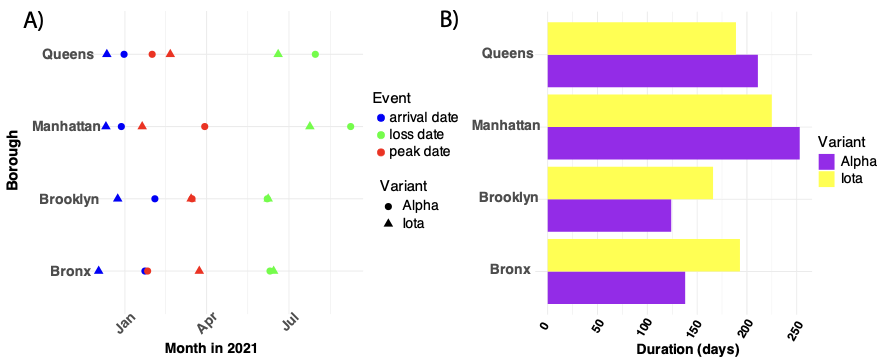


**Supplemental Figure 5:** Chronology of Alpha and Iota in Bronx, Brooklyn, Manhattan and Queens. **A)** Date of emergence (arrival date), Peak cases (peak date), and loss of detection (loss date) are plotted during 2021 to compare emergence patterns. **B)** The duration of the Alpha and Iota wave in days were calculated for each borough.


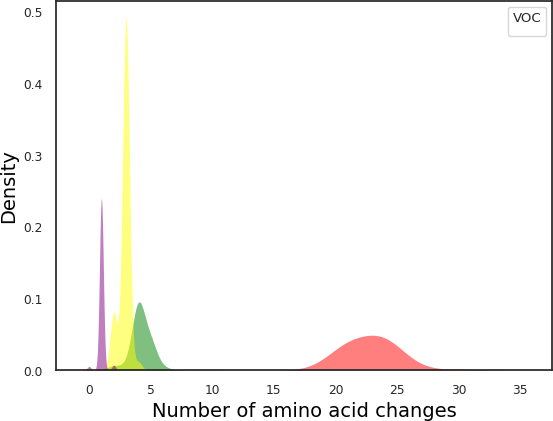


**Supplemental figure 6:** Density plot of antibody impacting mutations of Bronx Omicron, Delta, Iota and Alpha sequences form four statistically different distributions by a time-controlled linear model (p-value < 0.001).


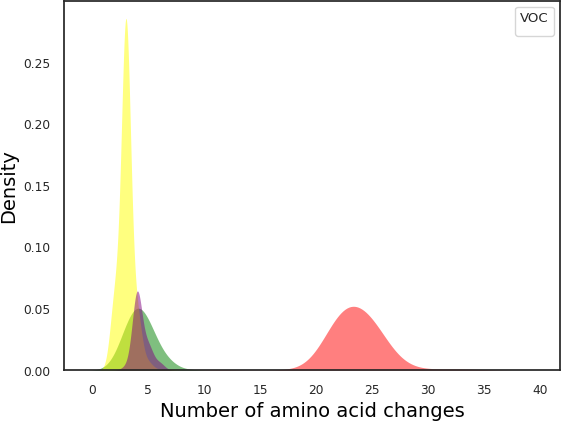


**Supplemental figure 7**: Density plot of t-cell epitope mutations show two statistically different distributions in the Bronx. Omicron forms it own distribution, where Delta, Iota and Alpha sequences form another distribution. Significance is assesed by a time-controlled linear model (p-value < 0.001).


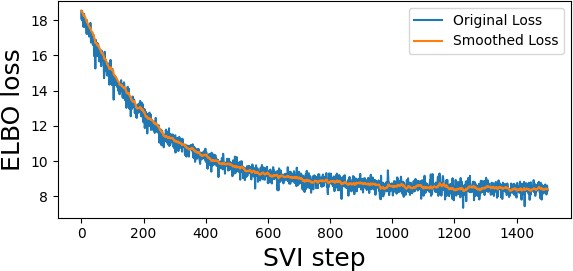


**Supplemental figure 8:** Example ELBO loss curve for bayesian logistic regression in local pyro model written in custom python script.

GISAID Identifier: EPI_SET_240811vt

doi: [10.55876/gis8.240811vt](https://doi.org/10.55876/gis8.240811vt)

All genome sequences and associated metadata in this dataset are published in GISAID’s EpiCoV database. To view the contributors of each individual sequence with details such as accession number, Virus name, Collection date, Originating Lab and Submitting Lab and the list of Authors, visit [10.55876/gis8.240811vt](https://doi.org/10.55876/gis8.240811vt)

# Data Snapshot

EPI_SET_240811vt is composed of 1,708 individual genome sequences. The collection dates range from 2020-03-04 to 2024-02-24;

Data were collected in 1 countries and territories;

All sequences in this dataset are compared relative to hCoV-19/Wuhan/WIV04/2019 (WIV04), the official reference sequence employed by GISAID (EPI_ISL_402124). Learn more at <https://gisaid.org/WIV04>.

GISAID Identifier: EPI_SET_240811wq

doi: [10.55876/gis8.240811wq](https://doi.org/10.55876/gis8.240811wq)

All genome sequences and associated metadata in this dataset are published in GISAID’s EpiCoV database. To view the contributors of each individual sequence with details such as accession number, Virus name, Collection date, Originating Lab and Submitting Lab and the list of Authors, visit [10.55876/gis8.240811wq](https://doi.org/10.55876/gis8.240811wq)

# Data Snapshot

EPI_SET_240811wq is composed of 2,142 individual genome sequences. The collection dates range from 2020-03-06 to 2024-02-26;

Data were collected in 1 countries and territories;

All sequences in this dataset are compared relative to hCoV-19/Wuhan/WIV04/2019 (WIV04), the official reference sequence employed by GISAID (EPI_ISL_402124). Learn more at <https://gisaid.org/WIV04>.

GISAID Identifier: EPI_SET_240811xa

doi: [10.55876/gis8.240811xa](https://doi.org/10.55876/gis8.240811xa)

All genome sequences and associated metadata in this dataset are published in GISAID’s EpiCoV database. To view the contributors of each individual sequence with details such as accession number, Virus name, Collection date, Originating Lab and Submitting Lab and the list of Authors, visit [10.55876/gis8.240811xa](https://doi.org/10.55876/gis8.240811xa)

# Data Snapshot

EPI_SET_240811xa is composed of 81,801 individual genome sequences. The collection dates range from 2020-03-04 to 2024-07-29;

Data were collected in 1 countries and territories;

All sequences in this dataset are compared relative to hCoV-19/Wuhan/WIV04/2019 (WIV04), the official reference sequence employed by GISAID (EPI_ISL_402124). Learn more at <https://gisaid.org/WIV04>.

GISAID Identifier: EPI_SET_240811zu

doi: [10.55876/gis8.240811zu](https://doi.org/10.55876/gis8.240811zu)

All genome sequences and associated metadata in this dataset are published in GISAID’s EpiCoV database. To view the contributors of each individual sequence with details such as accession number, Virus name, Collection date, Originating Lab and Submitting Lab and the list of Authors, visit [10.55876/gis8.240811zu](https://doi.org/10.55876/gis8.240811zu)

# Data Snapshot

EPI_SET_240811zu is composed of 1,129 individual genome sequences. The collection dates range from 2020-03-09 to 2022-09-10;

Data were collected in 1 countries and territories;

All sequences in this dataset are compared relative to hCoV-19/Wuhan/WIV04/2019 (WIV04), the official reference sequence employed by GISAID (EPI_ISL_402124). Learn more at <https://gisaid.org/WIV04>.
